# Role of AS-OCT in Managing Corneal Disorders

**DOI:** 10.3390/diagnostics12040918

**Published:** 2022-04-07

**Authors:** Nidhi Gupta, Akhil Varshney, Muralidhar Ramappa, Sayan Basu, Vito Romano, Manisha Acharya, Abha Gaur, Neha Kapur, Aastha Singh, Gaurav Shah, Isha Chaudhary, Nikunj Patel, Anil Tiwari, Anahita Kate, Virender Sangwan, Umang Mathur

**Affiliations:** 1 Department of Cornea, Dr. Shroff’s Charity Eye Hospital, New Delhi 110002, India; macharya@sceh.net (M.A.); abha@sceh.net (A.G.); neha.kapur@sceh.net (N.K.); aastha.singh@sceh.net (A.S.); gaurav.shah@sceh.net (G.S.); isha.chaudhari@sceh.net (I.C.); nikunj.patel@sceh.net (N.P.); anil.tiwari@sceh.net (A.T.); virender.sangwan@sceh.net (V.S.); umang@sceh.net (U.M.); 2 The Cornea Institute at L V Prasad Eye Institute, Hyderabad 500034, India; muralidhar@lvpei.org (M.R.); sayanbasu@lvpei.org (S.B.); dranahitakate@lvpei.org (A.K.); 3 Department of Medical and Surgical Specialities, Radiological Sciences and Public Health, Ophthalmology Clinic, University of Brescia, 25121 Brescia, Italy; vito.romano@unibs.it

**Keywords:** AS-OCT, cornea, ocular surface

## Abstract

Optical coherence tomography (OCT) is analogous to ultrasound biometry in the cross sectional imaging of ocular tissues. Development of current devices with deeper penetration and higher resolution has made it popular tool in clinics for visualization of anterior segment structures. In this review, the authors discussed the application of AS-OCT for diagnosis and management of various corneal and ocular surface disorders. Further, recent developments in the application of the device for pediatric corneal disorders and extending the application of OCT angiography for anterior segment are introduced.

## 1. Introduction

Anterior segment optical coherence tomography (AS-OCT) is a non-contact method of in vivo ocular imaging technology. It uses low coherence interferometry that measures the echo time delay of light backscattered from tissue structures and combines multiple axial scans into a composite B-scan image. Earlier ultrasound bio microscopy was used for anterior segment imaging, but with time AS-OCT has become a popular imaging tool for anterior segment imaging. AS-OCT is faster and provides more in-depth assessment of the anterior chamber and is repeatable and reproducible. For AS imaging, longer wavelengths (1050–1310 nm) are preferred than retinal OCT (800–900 nm) due to higher penetration and less scattering.

AS-OCT has been used to study various corneal pathologies such as keratitis, ecstatic disorders, corneal dystrophies and degenerations, and ocular surface disorders. There is preoperative planning and postoperative follow-up in refractive surgery, keratoplasty, and ocular trauma. The diagnosis of these disorders is predominantly clinical. However, clinical features may often be subtle or may even be subclinical and in such cases the use of AS-OCT helps clinch the diagnosis. Additionally, the OCT can also quantify the corneal changes which help grade the severity of the disease and assess its progression. The recovery occurring following therapeutic interventions can also be monitored with this device. The objective of the article is to highlight the important AS-OCT features of various corneal and ocular surface disorders.

## 2. Conjunctival Diseases

AS-OCT, also referred to as an optical biopsy, is an important non-invasive tool in diagnosis and monitoring of disease progression in various conjunctival diseases of the eye. Most notable of its uses is in differentiating malignant ocular surface squamous neoplasia (OSSN) from the benign lesions. The others uses are to differentiate a pterygium from a pseudopterygium and a pterygium from a pinguecula, to determine the precise measurement of a pterygium which helps to detect it’s growth and recurrence, and assess the risk of stromal scarring and astigmatism [1]. In a normal AS-OCT of the ocular surface, the tear film overlying the conjunctiva and the cornea is bright white. The corneal epithelium is thin and dark, while that of the conjunctiva and the limbus is thin but mildly hyper-reflective. The sub-epithelial tissue of the cornea is arranged in a regular linear pattern, whereas that of the conjunctiva is less regular and hyper-reflective.

**Pterygium:** A primary pterygium is seen as a hyper-reflective mass in the sub-epithelial conjunctival area extending into the cornea, causing elevation of the corneal epithelium and separating it from the underlying Bowman’s membrane. The pterygium tissue below the corneal epithelium extends beyond the corneal tip of the clinically evident pterygium mass, therefore, measuring the size of the pterygium more accurately.

**Pinguecula:** A Pinguecula is very similar to a pterygium but stops at the limbal region and doesn’t elevate the healthy corneal epithelium. There is also a clearly defined line of separation between the pinguecula and the underlying scleral tissue. 

**Pseudoterygium:** A pseudopterygium is seen as an overgrowing membrane that is not attached to the underlying cornea, with a plane of cleavage between it and the underlying corneal epithelium [2,3]. Increased risk of stromal scarring can be associated with pterygia with flat cornea-scleral transition zone as compared to the nodular variety, in pterygia with reduced apical head thickness, and in pterygia with partially disrupted Bowman’s membrane [1,2]. 

**Figure 1:**
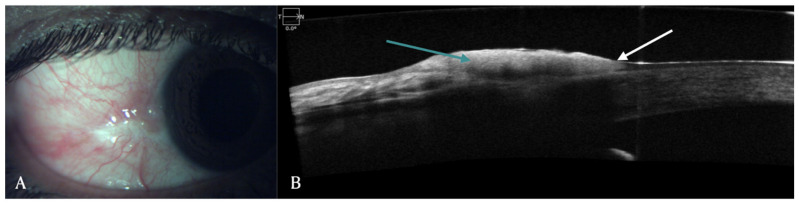
(**A**) Clinical picture of OSSN in a 51-year-old male patient. (**B**) Corresponding AS-OCT image showing thickened and hyper-reflective epithelial layer (green arrow) with abrupt transition from normal to abnormal epithelium (white arrow).

**Ocular surface tumors:** OSSN is a lesion involving epithelium of both cornea and conjunctiva. On AS-OCT imaging, OSSN can have either or all of these three characteristic features: hyper-reflective epithelium, thickened epithelial layer, and an abrupt transition from normal to abnormal epithelium (Figure 1) [4,5]. Another feature that has been identified in AS-OCT of OSSN cases is presence of hypo-reflective or cystoid spaces. These cystoid spaces could be type 1, which are small, round with hypo-reflective contents or type 2, which are larger, irregular with hyper-reflective material inside [6]. AS-OCT findings have a 94% sensitivity and 100% specificity for diagnosing OSSN and differentiating it from pterygium [7]. For OSSN treated with topical chemotherapy, AS-OCT after treatment showed normalization of epithelium [4,8]. Since AS-OCT can diagnose microscopic subclinical disease, it also prevents premature termination of topical chemotherapy in patients with residual subclinical disease and thus prevents the risk of recurrence [8,9]. AS-OCT helps in non-invasive diagnosis of OSSN and can be used to determine the need for treatment initiation as well as monitoring of disease course [4,5,10]. Since involvement of sub epithelial tissue and sclera is difficult in AS-OCT due to posterior shadowing of epithelial lesion, histopathology continues to be gold standard for diagnosis of deeper infiltration of tumor [10]. Angiographic features of OSSN have also been described using FA, ICGA and recently OCT-angiography has been used to demonstrate vasculature patterns. FA demonstrates vascular leakage while ICGA shows conjunctival feeder vessels and sea fan shaped intra-tumoral vessels [11]. OCT-A shows blood flow in epithelium and sub-epithelial tissues and highest density of vessels is seen in conjunctival part of tumor. It is also able to show corneal vessels under the tumor which are clinically not visible. Although OCT-A has the advantage of getting images without contrast dyes, compared to FA and ICGA, it cannot evaluate vascular leakage and feeder vessels [12]. In patients treated with topical chemotherapy such as 5-FU, FA, and ICG also demonstrated regression of vascular leakage, feeder vessels and intratumoral vessels after completion of therapy [11].

The conjunctival melanoma is seen as a hyper-reflective, sub epithelial lesion. A normal or slightly thickened epithelium is seen with hyper-reflective basal epithelium which suggests atypical melanocytes in the epithelium. This feature on AS-OCT helps in differentiating it from a pigmented OSSN [5,13]. The conjunctival naevus is seen as a well-circumscribed sub epithelial lesion underlying a normal epithelium. Cystic spaces are seen on AS-OCT, unlike melanomas. AS-OCT can also visualize cystic spaces in melanotic nevi seen in children, where cystic spaces are not clinically apparent [5]. AS-OCT images of conjunctival lymphomas typically show normal epithelium with homogenous, hypo-reflective (dark) sub epithelial lesions with smooth, well-defined borders. These can be differentiated from heterogenous, dark subepithelial lesions with irregular, diffuse borders seen in cases of conjunctival amyloidosis [5,13]. 

## 3. Limbal Stem Cell Deficiency (LSCD)

LSCD on OCT manifests as thinning of the limbal and corneal epithelium [14,15]. These changes are global in cases of total LSCD while in partial disease they are more profound in the affected areas. The unaffected areas may have normal epithelial thickness or it may be reduced, indicating a subclinical form of the disease [14]. Additionally, direct visualization of the disrupted palisades of Vogt is possible with the use of this device [16]. With the use of image processing software, Varma et al., have described a method of quantifying the reflectivity patterns of the line scans of OCT and both epithelial and stromal reflectivities were studied in eyes with LSCD. The authors devised a ratio between them (ES ratio) and proposed that values greater than 1.29 were diagnostic of LSCD. Several corneal disorders with vascularization may mimic LSCD. With the help of the segmentation feature of the OCTA, differentiating the true LSCD cases is possible as the vasculature in LSCD is usually restricted to the superficial layers [17]. A superficial vascular density value greater than 0.38 has good sensitivity and specificity for LSCD [17].

**Figure 2:**
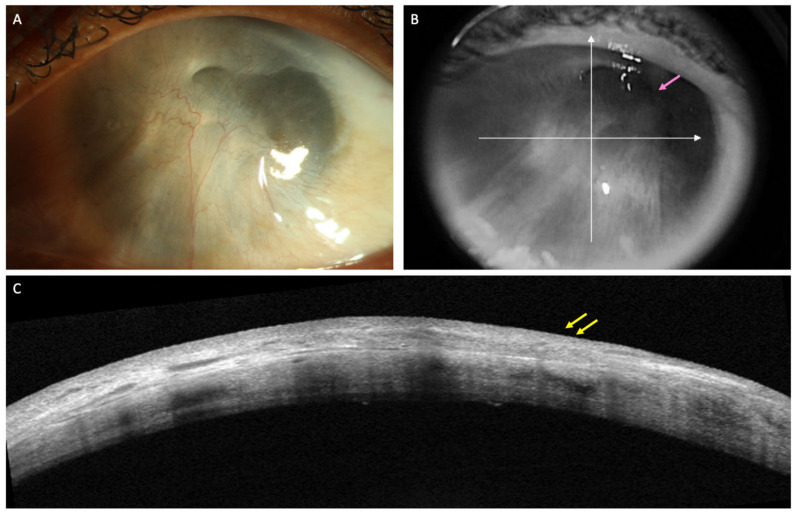
(**A**–**C**) Clinical images of the left eye of a patient with limbal stem cell deficiency (LSCD) due to chronic sequelae of ocular chemical burns. (**A**) Slit lamp image depicting total limbal stem cell deficiency with a thick fibrotic pannus covering the visual axis. (**B**) Details of the anterior chamber can be hazily visualized in the superotemporal quadrant (pink arrow) in the infrared image of optical coherence tomography (AS-OCT) scan. (**C**) AS-OCT line scan showing the hyperreflective pannus (yellow arrows) with sufficient corneal stromal thickness to permit pannus dissection.

**Figure 3:**
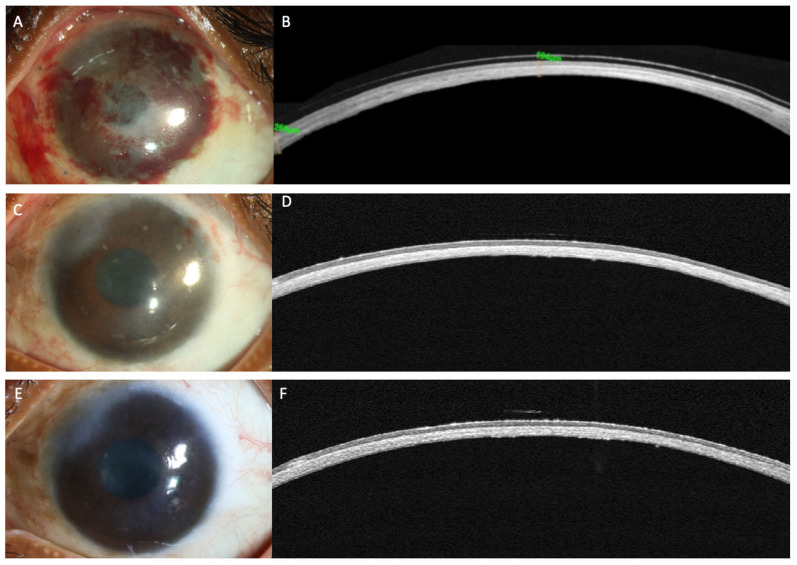
(**A**–**F**) This is a collage of images depicting recovery following simple limbal epithelial transplantation (SLET) in the patient from Figure 2. (**A**,**B**) Slit lamp image and the corresponding optical coherence tomography (OCT) image captured in the immediate postoperative period illustrating thinning of the corneal stroma with absence of the hyperreflective conjunctival epithelium. (**C**) The surface is well epithelialized, six weeks after SLET with stromal thinning evident on the OCT scan along with a normal hypo-reflective corneal epithelium (**D**) The stable ocular surface is maintained 5 months after the surgery (**E**,**F**).

Limbal stem cell transplantation (LSCT) is perhaps one of the most popular treatment options in eyes with LSCD. Of the different types of LSCT, simple limbal epithelial transplantation (SLET) is preferred for both unilateral and bilateral LSCD in view of its single-step technique which does not require any additional laboratory support and yields stable results [18]. An important step of SLET is the dissection of the overlying fibrovascular pannus. However, the pannus may preclude assessment of the underlying stromal thickness and scarring on clinical examination and so perforation of the cornea may occur during this step in very thin corneas. Preoperative assessment of stromal thinning on OCT may help identifying such cases (Figure 2). The degree of stromal opacification may also be visible on these scans and the need for a secondary intervention for visual rehabilitation can be planned accordingly. The infra-red image of the cornea that accompanies the line scans also provides valuable information in the decision making in such cases (Figure 3). If the anterior chamber details are discernible, performing SLET in isolation is usually sufficient while a keratoplasty may be required if no structures are visible [18,19]. The postoperative monitoring of recovery of the corneal epithelium following SLET is also feasible with an AS-OCT (Figure 3) [20]. The ES ratio gradually decreases and returns to near normal values and this trend can be followed up with AS-OCT scans [20].

## 4. Ocular Chemical Burns (OCBs)

**Figure 4:**
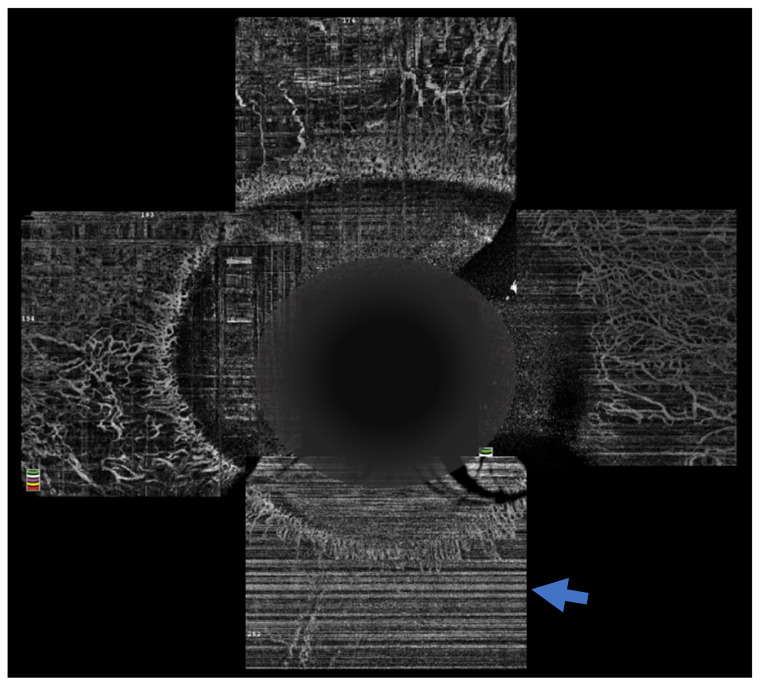
Optical coherence tomography angiography of an eye with chemical injury illustrating lack of vasculature inferiorly (arrow), in the area with ischemia.

The sequelae that ensue from OCBs usually depend on the severity of injury in the acute phase of the disease. One of the most important clinical findings that helps prognosticate these eyes is the presence of limbal ischemia. However, detection of this finding in the acute phase is subjectable and often incorrectly gauged [21]. OCTA provides an objective way of assessing the presence of and quantifying the extent of limbal ischemia (Figure 4). The extent of ischemia as measured by an OCTA is comparable to the ischemia assessed by standard angiography techniques [22]. Various indices have been described of which vessel density is most frequently used and correlates with the severity of the injury [23,24]. Incorporation of these parameters in the existing grading systems is being attempted to provide a more accurate method of predicting the prognosis of these cases [23]. Furthermore, the need for interventions in the acute phase, such as a tenonplasty, can also be determined by the severity and location of the ischemia. The degree of ischemia present may also predict the rate of epithelial healing and these eyes can be closely monitored for the need for procedures such an amniotic membrane grafting to augment the healing process. OCTA was used for serial monitoring of the suspect areas can help confirm the presence of ischemia, as vasospasm is reversible, and the normal vasculature becomes evident on the subsequent scans [25].

## 5. Dry Eye Disease (DED)

The use of AS-OCT for DED has primarily been described for the assessment of the tear film meniscus. This evaluation is carried out in terms of the tear film height, area, and depth. Of these parameters, the area of the meniscus correlates the best with traditional DED tests such as Schirmer and tear film breakup time tests and can diagnose DED with good sensitivity and specificity [26,27]. The non-contact nature of the AS-OCT is an added advantage over routine diagnostic tests for DED. Furthermore, the Schirmer values can be affected by several factors such as the surrounding environment, the prior use of medications, the placement of the strip, etc. [27,28]. The AS-OCT in contrast is more reliable with good repeatability of the derived values [27,29]. The height of conjunctival folds and extent of tear film covering the same has also been studied with AS-OCT in DED and can be used as an objective method of DED evaluation [29]. The device can also help to monitor response to therapy with topical medications and punctal occlusion [30].

## 6. Keratoconus

AS-OCT serves as an adjunct to clinical diagnosis in cases of keratoconus, where the epithelium is seen as thinned out over protruding stroma and displays an annular thickening over the surrounding flatter stroma leading to a doughnut configuration [31]. In addition, a higher variability of epithelium thickness measured by pattern standard deviation is also noted in keratoconus [32]. Increased epithelial thickness and stromal thinning at the corneal apex; and a hyper-reflective Bowman’s layer serves as the risk factors in predicting acute hydrops [33,34]. The severity and extent of Descemet’s membrane detachment (DMD) as measured on AS-OCT serves as an additional advantage to clinicians to predict the resolution and treatment with air/gas injection post-acute hydrops. The depth of treated tissue post-CXL is easily assessed using the demarcation line on AS-OCT.

In eyes treated with traditional Dresden protocol, the depth of the demarcation line is approximately 300 μm [35] and shallower in accelerated CXL. AS-OCT also helpful in the evaluating the depth of implantation of intrastromal ring segments [36,37]. 

## 7. Corneal Dystrophy

Before the advent of AS-OCT, slit lamp examination (SLE) was the only tool to determine the morphological changes in different dystrophies. A histopathological examination was only possible after surgical removal of the tissue. AS-OCT allows for morphologic differentiation of different dystrophies with instantaneous high-resolution images, even in irregular and opaque corneas. With its near histological resolution, AS-OCT allows enhanced evaluation of structural changes and plays a role in clinical decisions for selecting the surgical technique best suited for the removal of corneal debris and scar tissue [38,39]. In Fuch’s endothelial corneal dystrophy (FECD), the corneal central-to-peripheral thickness ratio (CPTR), measured by the AS-OCT, is reported to be an objective and repeatable measure of severity of FECD [40]. It is also a helpful tool in evaluating and documenting progression of the dystrophies [39].

## 8. Infective Keratitis

The precise clinical diagnosis, accurate diagnostic tools, and timely appropriate management are important to reduce the morbidity associated with infectious keratitis. Slit-lamp biomicroscopy is the routine instrument used for the clinical examination of keratitis to assess the site and size of infiltrates and epithelial defect. Measuring the depth of the corneal infiltration and thickness of cornea is not very accurate with a slit lamp. Since AS-OCT provides cross-sectional scans of the cornea, it helps to evaluate the depth of stromal infiltration and corneal thickness and many ulcer characteristics can be assessed with AS-OCT. 

Epithelial defect is seen as breach in the topmost hyper-reflective layer in normal areas. The edges of the ulcer can be sloping or straight or undermined, and the bed of the ulcerated area can be seen as irregular with ill-defined margins [41,42]. Corneal perforation can be seen as a localized area of stromal thinning with irregular hyperreflective area (uveal tissue) prolapse noted in the area of the defect. Infiltrate is seen as hyperreflective area with ill-defined margins. It can have posterior shadowing with an overlying epithelial defect, healing of ulcer is seen as hyperreflective area (scar) with healed epithelial defect [43]. There is thinning of cornea and appearance of a demarcation line. Resolution of corneal infection is characterized by an early reduction in corneal edema, followed by a later reduction in infiltration: both parameters can be routinely measured and followed with AS-OCT, helping in the monitoring of the pathology and the response to the treatment [44]. Satellite lesions are seen as hyperreflective area with posterior shadowing separate from a central hyperreflective area (infiltrate). An intense hyperreflective areas overlying the epithelial defect could be either drug deposits or epithelial plaques or pigmented plaques [42]. Necrotic areas are identified as the presence of cystic spaces. Corneal edema has diffused thickening of the stroma, change in the convexity of the posterior corneal surface.

In patients with acanthamoeba keratitis (AK), radial keratoneurites can be seen with AS-OCT as highly reflective lines or bands of different width which run into the corneal stroma obliquely or parallel. Yamazaki et al. [45] found that such lesions become thinner and eventually disappear after the resolution of the keratitis. Soliman et al. [46] observed that in cases of herpes simplex keratitis, corneal infiltrates are seen on AS-OCT as a lentiform or spindle-shaped hyper-reflective area in the stroma that may be diffuse or localized, although these were not specific. In corneal endothelitis, coin-shaped lesions are imaged on AS-OCT as an irregularly highly reflective thickened endothelium, as described by Yokogawa et al. [47]. Multiple whitish, raised, epithelial lesions of microsporidiosis can be seen as raised hyper-reflective epithelial lesions [48]. AS-OCT might help in differentiating them from adenovirus nummular scars which are subepithelial and not epithelial lesions. In clinical practice, serial standardized AS-OCT scanning might be used to quantify and objectively assess the keratitis. 

AS-OCT also has various limitations. In case of dense infiltrates, depth of the infiltrates and corneal thickness (CT) cannot be measured accurately because of posterior shadowing. However, they can be used to monitor response. It is difficult to differentiate among drug deposits, epithelial plaques, and pigment deposition on AS-OCT because all are seen as hyperreflective areas overlying the epithelial defect. It does not measure the density of infiltrates which can be a valuable parameter in the follow-up of corneal infections.

## 9. Corneal Transplantation

**Figure 5:**
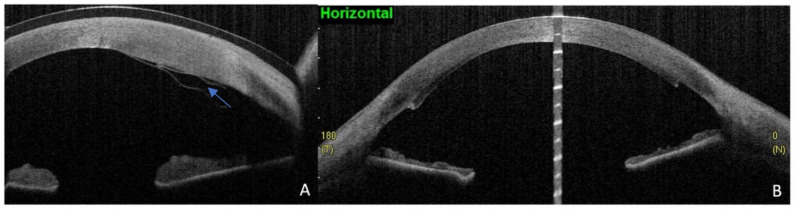
(**A**) Early postoperative AS-OCT scan shows a DMEK graft fold as indicated by blue arrow; (**B**) postoperative AS-OCT scan UT-DSAEK where is possible to evaluate graft thickness and profile.

AS-OCT is becoming a very useful tool for planning and monitoring patients of corneal transplantation. AS-OCT is useful in planning endothelial keratoplasty to investigate in detail the status of the anterior segment and planning a DSAEK, DMEK or PK (Figure 5). Specifically useful to know the morphology or posterior cornea and the smoothness of the posterior cornea especially in case of DMEK. It helps in detection of corneal scarring and/haze, presence of synechias, tube, and iris defects [49]. AS-OCT plays also a key role in DSAEK preparation, where achieving results consistently can be challenging as this procedure relies on microkeratome cutting. It may result in a thick graft and irregular graft profile hampering the patient’s visual quality and go unnoticed until post-operative poor vision is encountered. The AS-OCT allows to assess the DSAEK lenticule following microkeratome pass, ensuring its profile is uniform and its thickness is under 100 microns [50,51]. Handheld ultra sound pachymetry in graft preparation may help with the microkeratome head selection, but it is unreliable at providing graft details (graft thickness and profile) once the microkeratome pass is made. Eye banks also uses AS-OCT providing to surgeons a fully validated tissue giving graft profile, details of corneal thickness, and endothelial cell counts [52].

AS-OCT in endothelial keratoplasty is also useful for immediate identification of graft detachment [53,54,55,56,57,58,59,60]. Comparing AS-OCT, Scheimpflug imaging and slit-lamp biomicroscopy for the detection of DMEK graft detachment, Moutsouris et al., reported that AS-OCT was superior to Scheimpflug imaging in confirming the diagnosis of graft attachment/detachment in 36% of eyes in which conclusive diagnosis could not be made by slit-lamp microscopy alone [56]. Identification and quantification of graft detachment is critically important post-EK and DMEK as a decision must be made on the necessity for repeat bubble injection for graft repositioning and tamponade.

AS-OCT plays also a rule in anterior lamellar surgery. To maximize the success rate of pneumatic dissection, before injecting air, the needle/cannula should reach the central cornea as close as possible to the posterior corneal stromal surface. Scorcia et al., using an intraoperative optical coherence tomography confirmed that the rate of big bubble formation exceeds 90% when the needle/cannula reaches a depth within 100 microns from the internal corneal surface [61]. Between all of the imaging technology described to assist the pneumatic dissection the more promising seems to be the optical coherence tomography, its advantage versus the ultrasound pachymetry is evident in very thin corneas and in localizing areas of thinning and scarring. There is also on-going research aim to design surgical instrument that do not obstruct image acquisition of the OCT. However, it must be highlighted that its costs and its impact on surgical time is a concern [62]. AS-OCT explains its function especially in planning the keratoplasty with the femtolaser [63,64,65].

## 10. Refractive Surgery

The AS-OCT serves as a very useful tool in the planning of both kerato-refractive surgery, phakic IOLs, and cataract surgery. In planning of phakic IOLs its essential to know the anterior chamber depth, and also the predicted positioning of the IOL with respect to the corneal endothelium which is very accurately obtained on the AS-OCT [66]. Baikoff et al., on serial AS-OCT have successfully predicted how the changes in the natural lens with age also affect the positioning of the phakic IOL, hence avoiding any post operative complications [67]. 

In the keratorefractive procedures such as LASIK, the high-resolution images of AS-OCT are important to evaluate post-operative complications of residual myopic error and in their management. The images obtained very accurately measure the flap thickness and the residual stromal bed and can help predict chances of ectasia in cases of enhancement procedures [68]. Also, this device has been used extensively to evaluate the architecture of the femtosecond flaps and the microkeratome flaps and document the advantages of one over the other. Zhang et al., have successfully demonstrated that femtosecond LASER creates significantly more uniform and reproducible flaps [69]. 

Another post-operative application of AS-OCT, in corneal flap-based procedures is the detection of interface fluid syndrome (IFS) which usually presents in the early post operative period but has been documented even after two years post-surgery [70]. IFS post LASIK is a rare but potentially sight threatening complication because of increase in the intraocular pressure. Significant rise in IOP causes exudation of fluid in the cornea due to an imbalance in the endothelial pump, this gets collected in the flap bed interface presenting as an edematous flap and often mimics diffuse lamellar keratitis (DLK). AS-OCT helps in differentiating the two conditions as the management of either is very different, and applanation tonometry also may sometimes be doubtful [71]. High dose steroids given for DLK further worsens IFS where AS-OCT imaging plays an important role is the correct diagnosis of IFS. 

## 11. Pediatric Corneal Disease

AS-OCT provides invaluable information in diagnosis, prognostication, grading, assessing surgical outcomes, and deciding on the lines of management required in a wide range of developmental and or acquired abnormalities of the cornea and anterior segment in children. AS-OCT’s value is its ability to give higher resolution structural information through even opaque corneas, objective grading of corneal transparency using light backscattering, measuring anterior chamber parameters, including angle scans, pachymetry, depth of the lesion, and secondary changes due to longstanding cornea edema that further help in the better surgical management of these conditions. Hand-held AS-OCT can be used without the need for general anesthesia. Furthermore, AS-OCT provides in-vivo, real-time anatomical ultrastructure details in cross-sectional and segmental visual presentation.

Therefore, AS-OCT imaging is crucial in understanding the complex nature of these disorders.

Congenital corneal opacities: these are a group of complex heterogeneous disorders with overlapping phenotypic similarities on presentation and are often challenging to diagnose and ascertain surgical outcomes. AS-OCT can elicit more detailed structural findings, thus aiding in accurate diagnosis.

**Figure 6:**
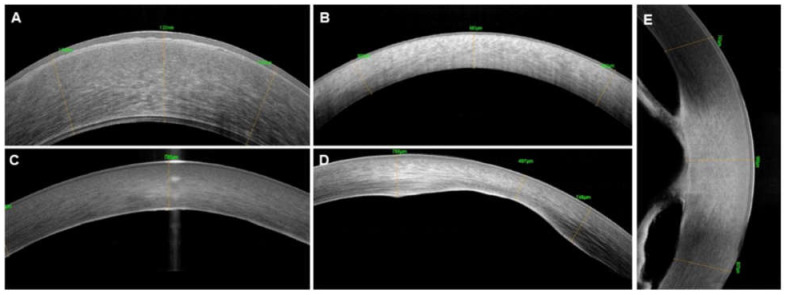
**Anterior segment optical coherence tomography images of various corneal conditions.** Congenital hereditary endothelial dystrophy (CHED)—a thickened epithelial layer with underlying irregular bowman’s membrane, increased stromal thickness and abnormally thickened Descemet’s membrane (**A**); Hurler’s syndrome—a thickened corneal stromal layers and hyperreflective stroma (**B**); posterior polymorphous corneal dystrophy (PPCD)—abnormal thickening and hyperreflective Descemet’s membrane (**C**); Peters Anomaly—posterior corneal defect (**D**); and kerato-irido-lenticular contact (**E**).

**Peters’ anomaly (PA)** is characterized by posterior corneal excavation with a focal absence of endothelium and Descemet’s membrane with a posterior stromal opacification and further categorized based on presence of iridocorneal adhesions in type-1 PA (Figure 6) or Kerato-lenticular insertion as seen in type-2 PA and pan corneal involvement with staphylomatous changes seen in severe spectrum [72]. AS-OCT can help in accurate diagnosis, ascertaining the type, severity, and grading of PA, thus predicting the surgical success of keratoplasty. Children diagnosed with PA should be imaged using AS-OCT. When available, high-resolution images provide insight into the microstructure of the anterior segment and help in categorizing PA and for surgical decision making, thus preventing unnecessary surgeries or intraoperative iris, and lenticular injuries.

**Sclerocornea** is a congenital, non-progressive, non-inflammatory condition involving one or both corneas, showing some degree of opacification and flattening corneal curvature. Typically, peripheral corneal is involvement with a limbal abnormality and corneal opacity with an obscured border between the sclera and cornea. AS-OCT can be used in quantifying the limbal palisades and help prognosticate keratoplasty outcomes [73]. However, a considerable overlap does exist between Peters anomaly and sclerocornea in the nomenclature and the clinical description, AS-OCT provides a clearer insight in such cases.

**Congenital corneal staphyloma** has been presented as an unusual variant of either PA or sclerocornea characterized by anterior bulging of ectatic cornea and pigment epithelium cell lining of the posterior corneal surface.

**Primary congenital aphakia:** Congenital primary aphakia [74] can be either an isolated abnormality or a part of complex anterior segment abnormality, including, microphthalmia, absence of the iris, anterior segment aplasia, and/or sclerocornea, or it may occur in association with multiple somatic abnormalities. It is of vital importance to assess the lens status via either–B scan, Ultrasound Biomicroscopy (UBM) or OCT in cases where corneal involvement precludes the assessment of the anterior and posterior segment, before performing a surgical procedure. This is to rule out associated comorbidities that can directly influence the surgical outcomes.

Congenital hereditary endothelial dystrophy (CHED) [75] with its typical increase in corneal thickness and thickened epithelial layer with underlying irregular bowman’s membrane, increased stromal thickness, and abnormally thickened hypo-reflective Descemet’s membrane (Figure 6) can be differentiated from primary congenital glaucoma (PCG) or birth trauma which will have an average corneal thickness with the presence of horizontal or vertical/oblique lines of Descemet membrane rupture (Haab’s striae) respectively. Also, AS-OCT unravels anterior stromal secondary changes, particularly in children with longstanding cornea edema, thus aiding prognosis of surgical outcome and choosing the most appropriate surgical strategy, meaning situations where PKP outscore DSAEK/DMEK owing to a significant degenerative pannus, anterior stromal haze, or opacification. After a pediatric DSAEK, OCT helps ascertain lenticular status including orientation, monitor long-term graft host dynamics, and recognize allograft rejection or graft failure early.

**Mucopolysaccharidosis (MPS):** AS-OCT allows objective assessment of anterior segment architectural changes in children with cloudy cornea due to MPS. The estimated prevalence of secondary glaucoma in cases of MPS was 2.1 to 12.5%. The severe ocular spectrum is likely to have a higher propensity due to thickened uveal tissues, shallow anterior chamber structure, and accumulation of glycosaminoglycans (GAGs) within the trabecular meshwork. Zhang and colleagues earlier reported glaucoma in MPS type I, IV, VI cases. In our ongoing study, anterior chamber depth (ACD) (2.99 ± 0.29) in MPS was smaller than ACD (3.58 ± 0.17) of age-matched normal. AS-OCT may provide the basis for the early onset of secondary glaucoma independent of the optic nerve or visual field changes. It may also facilitate follow-up examinations and enable objective evaluation of disease progression in MPS.

**Trauma:** AS-OCT useful in angle assessment in the pediatric age group particularly in cases of anterior segment trauma. Since it is non-invasive, OCT can be used safely in situations in which ocular tissue has been lacerated or punctured, conditions under which gonioscopy cannot be performed because owing communicating wound with an aqueous leakage.

**Implantation corneal and iris cyst:** Iris cysts can be divided into primary cysts and secondary cysts based on a classification proposed by Shields et al. [76]. Primary cysts include cysts of the iris pigment epithelium or cysts of the iris stroma that are mostly stationary, rarely progress or cause significant visual complications. Acquired cysts occur secondary to epithelial downgrowth either following trauma or surgery or secondary to tumors, parasitic invasion of the anterior chamber or after prolonged usage of topical medications such as miotics or prostaglandin analogues. In such cases, AS-OCT can provide useful diagnostic aid in ruling out various presentations of the above that may appear to masquerade as something else. Also, the size, location, extent, and presence of a fistulous tract can be readily visualized pre-operatively [76].

**Microscope Integrated Optical Coherence Tomography (Mi-OCT):** This could provide real-time, high-resolution, cross-sectional images during various surgical steps [77]. This device is invaluable during intraoperative maneuverability, particularly in children with cloudy corneas undergoing selective corneal replacement. For instance, the double-ring sign intraoperatively confirms correct lenticular orientation, but in cases of the cloudy cornea, Mi-OCT is useful in visualizing acutely angled bevel sign and could be helpful in confirmation of the graft orientation and attachment as well. Besides this, it also helps in recognizing anterior and or posterior synechia, iris or lens corneal touch, thus minimizing intraoperative complications.

In conclusion, published literature have demonstrated utility of real-time imaging of the pediatric anterior segment using high-speed AS-OCT for diagnosis and surgical planning. Fast data acquisition allows real-time display of high-quality images in which delineation of the corneal layers and full-thickness visualization of angle structures is possible. Measurements of corneal epithelial and stromal thickness and of the anterior chamber angle are made. These results suggest the potential use of real-time AS-OCT in the clinical assessment of the anterior segment, especially as an adjunct in glaucoma evaluation and for intraoperative monitoring of corneal changes during corneal transplantation. 

## 12. Corneal Trauma and Foreign Body

AS-OCT can be used as an adjunct to routine slit-lamp biomicroscopy in ocular trauma cases as a noncontact modality in otherwise fragile traumatic eyes. In patients with corneal edema after blunt ocular trauma, AS-OCT helps in diagnosing underlying Descemet’s detachment which can be missed with routine slit-lamp examination [78]. In patients with hyphemia, corneal blood staining has been described as zone of hyperreflectivity in the posterior stroma indicating presence of red blood cell products. Any loss of this hyperreflectivity at Descemet’s membrane (DM) level on serial scans indicates permanent damage and obviates the need of endothelial keratoplasty in the future [78].

In patients with penetrating injury, the differentiation of full-thickness tears from lamellar corneal lacerations can be noted. AS-OCT can be also used predict healing response after primary repair of corneal tear [79]. The internal step height immediately after the repair is a very good predictor of scar intensity and outcome at six months. Step height less than 80 µm is found to heal faster and majority of the wound edges would oppose between three and six months [80]. Wounds with adherent intraocular tissue would take longer time to heal and are associated with higher corneal thickness and poor visual outcomes.

Often corneal foreign body type and depth cannot be clearly discerned on slit-lamp examination, and aggressive interventions to remove deep corneal foreign bodies may result in corneal perforation. AS-OCT can be useful in such conditions [81]. Anterior border of opaque objects (metal, wood, pencil graphite) are found to be hyperreflective, whereas the posterior border signal cannot be clearly identified due to the shadowing effect. Mirroring effect (inverted chain of signals) is observed with metal and pencil graphite. Transparent materials demonstrated hyper-reflective sharp borders when surrounded by air or fluid, as opposed to when being embedded purely in the corneal stroma. Organic foreign bodies (e.g., chestnut burr) does not have any characteristic findings on AS-OCT [82]. 

AS-OCT provides vital details about Descemet’s membrane integrity and the point of entry of a foreign body, which can be utilized to plan surgical removal. When Descemet’s membrane is intact, the foreign body is removed via the anterior surface route, whereas when Descemet’s membrane is breached and the foreign body point of entry was healed the foreign body is removed via the anterior chamber [83]. AS-OCT can also be utilized in diagnosing retained intraocular foreign body masquerading as chronic anterior uveitis [84].

## 13. AS-OCT in Animal Experiments

In wound models, AS-OCT has been used to assess the depth of wound creation (anterior- mid or posterior stromal) and corneal thickness or pachymetry in order to assess wound remodeling and scarring. Novel method to document stromal opacity on AS-OCT in terms of epithelial to stromal (E:S) reflectivity ratio has been reported by Joshi et al. [85]. AS-OCT has been used to monitor endothelial disruption, iridocorneal adhesion in experimental mouse model of bullous keratopathy by cryoinjury [86]. Additionally, it has been documented in experimental models of microbial keratitis such as HSV and dry eye. Ultrahigh- resolution micro-OCT have been shown to detect inflammatory cells invading the corneal endothelium and stromal layers [87].

In the regenerative and bioengineering realm, AS-OCT has been used to demonstrate retainability and stability of hydrogels. Promising results can help us develop these modalities as a possible treatment alternative to corneal transplantation in patients of debilitating corneal injury and scarring [88]. 

AS-OCT has been used to assess graft fate in rabbit and monkey models of corneal endothelial cell dysfunction by measuring corneal thickness and keratic precipitates as an indication of the inflammatory reaction. The findings of the study have been used to propose measurement of central corneal thickness as a reliable tool to measure graft success [89]. 

Animal models have also been used to upgrade algorithms in the machine itself. Newer algorithms such as ultrahigh-resolution (UHR)-OCT, which has been shown to provide precise assessment of epithelial wound healing and 3-dimensional display in rabbit model and super luminescent diode array based in vivo micro-OCT system which has reported to detect pathology in the presence of corneal scarring can help in widening its clinical use [90,91].

## 14. AS-OCT Angiography

A variety of disease response such as inflammation, infection, degeneration, and trauma could disturb the ocular surface balance and leading to corneal neovascularization (CNV). CNV compromises the transparency of the cornea resulting in poor vision and finally loss of vision [92,93]. The most common way of assessing anterior segment vasculature is through slit-lamp photography (SLP) and fluorescein dye-based angiography. Though widely used, SLP has certain limitations such as poor visualization of blood-vessels in the presence of corneal edema or in fibrotic cornea, resulting in compromised image analysis and poor sensitivity for the smaller vessels [94]. With the recent advancement in image acquisition and analysis technology, OCT uses has recently increased in visualizing and analyzing blood vessels too resulting in OCT angiography (OCTA), an emerging method for imaging ocular vasculature [95,96]. Though OCTA is widely used in posterior segment imaging, its application for the anterior segment is poorly explored. AS-OCTA is a new technology to visualize anterior of the eye blood vessels. It has immense potential for future clinical diagnosis and applications in corneal and iris pathologies, pre-operative surgical planning, evaluating the efficacy of new antiangiogenic drugs and assessing limbal stem cell deficiency [95,96,97,98].

A recent pilot animal model study highlights the importance of AS-OCTA as a useful non-invasive imaging tool for objective assessment of CNV for examining the efficacy of a drug on the angiogenesis., which is essential for further clinical studies [99]. In clinics AS-OCTA has shown to be a promising tool for CNV, LSCD and to detect subclinical neovascularization of iris (NVI) that appeared in its early stages.

Advancement of technology and convergence of health care and computational biology, it is compelling to use artificial intelligence to produce normative databases and carry out deep analysis for appropriate AS-OCTA studies. Further studies are needed for designing the feasibility of combining OCTA with fluorescence angiography using multi-modal approaches. With further development, OCTA for anterior segment imaging in the clinics may become common in the near future.

## 15. Conclusions

AS-OCT is a noninvasive imaging tool that provides accurate and reproducible images of cornea and ocular surface. It is increasingly becoming popular with improving technology to achieve better resolution and helping clinicians to come to a definitive diagnosis by correlating the suspicious lesions with AS-OCT images. Therefore, applications of AS-OCT in evaluation of cornea and ocular surface disorder have greater potential in clinical practice, although there is still limited use of this tool by clinician in their clinical practice. The goal of this review is to describe the AS-OCT features in variety of cornea and ocular surface disorders of human patient and experimental animal model with representative figures of AS-OCT images. The information in this paper will help clinicians to use AS-OCT for diagnoses and surgical planning of the patients. Future studies are needed to extend the use of AS-OCT in managing cornea and ocular surface (with emerging technology of OCTA as well).

## Data Availability

Not applicable.
